# Evolution of Management of Postoperative Pain in Spain: A Nationwide Survey of Organizational Structure and Clinical Practice

**DOI:** 10.1155/prm/5391303

**Published:** 2026-11-02

**Authors:** Maria A. Perez-Herrero, Gustavo Illodo Miramontes, Neus Esteve Pérez, Servando López Alvarez, Juan Carlos Luís Navarro, Blanca Martínez Serrano, Santiago Montesinos Fadrique, Sandra Fernández Caballero, María Teresa Fernández Martin, Agustín Mendiola de la Osa, Antonio Montes Pérez

**Affiliations:** ^1^ Department of Anaesthesia and Reanimation Hospital Clínico Universitario Valladolid Spain saludcastillayleon.es; ^2^ Department of Anaesthesia and Reanimation University Complex of Vigo Vigo Spain; ^3^ Department of Anaesthesia, Reanimation and Pain Management Hospital San Joan de Déu Palma Islas Baleares Spain; ^4^ Department of Anaesthesia, Reanimation and Pain Management Complexo Hospitalario Universitario A Coruña Spain; ^5^ Department of Anaesthesia Hospital Quironsalud Sagrado Corazón Sevilla Spain; ^6^ Department of Anaesthesia, Reanimation and Pain Management Hospital Universitario La Paz Madrid Spain hulp.es; ^7^ Department of Anaesthesia, Reanimation and Pain Management Hospital Arnau de Vilanova Valencia Spain esmo.org; ^8^ Department of Anaesthesia, Reanimation and Pain Management Hospital Universitario Río Hortega Valladolid Spain saludcastillayleon.es; ^9^ Department of Anaesthesia, Reanimation and Pain Management Hospital Puerta de Hierro Majadahonda Majadahonda Spain; ^10^ Department of Anaesthesiology Hospital Del Mar Research Institute Barcelona Spain imim.cat

**Keywords:** data collection, healthcare surveys, hospitals, pain, postoperative, surveys and questionnaires

## Abstract

**Background:**

Hospitals require standardized processes to effectively manage patients with acute postoperative pain. We aim to evaluate the current pain management practices in Spain through a cross‐sectional study, updating the findings from a previous audit conducted in 2012.

**Methods:**

An online questionnaire was mailed to anesthesiologists in Spain. Responses were stratified based on the hospital size (< 200 or ≥ 200 beds). Categorical variables were presented as percentages with a 95% confidence interval, while continuous variables were expressed as median and interquartile range.

**Results:**

The survey received responses from 67.9% (*n* = 127) of hospitals with ≥ 200 beds. 70.1% of large hospitals had an acute pain service (APS) and a hospital‐wide acute pain management program (APMP). Concerning large hospitals, full‐time pain physicians were available in 30.3% of large hospitals, and 41.6% had full‐time nurses dedicated to pain management; 40.2% of patients received written information about postoperative pain; 89.0% of participants reported that their hospital used a standardized scale for assessing postoperative pain; 79.5% had pain treatment protocols; professional training in postoperative pain was organized in 46.6% of cases; and 51.2% considered that the acute postoperative pain was well managed in their hospital.

**Conclusion:**

Twelve years later, our study shows (in hospitals ≥ 200 beds), there has been an increase in the percentage of centers with APS or APMP, the proportion of nurses with full‐time dedication, and the assessment and documentation of pain. However, the implementation of protocols, pain management training, and the analysis of pain‐related indicators have worsened. Improvements are necessary to implement standardized acute postoperative pain management.

**Significance statement**
 It is necessary to define, at the national level, solid structures in the management of acute postoperative pain, which allow for reducing the existing variability.

## 1. Introduction

Optimal prevention and relief of postoperative pain are essential on humanitarian grounds and for the efficient delivery of healthcare services [1]; although several analgesic therapies are available, effective postoperative pain management is a clinical challenge [2]. Specialist teams for managing postoperative analgesia emerged in the 1970s to centralize responsibility for pain control. Since then, several evidence‐based guidelines for perioperative pain management have been developed [3–5]. However, these recommendations have not yet been widely adopted, and it is difficult to determine how effectively acute pain services (APSs) achieve their goals in the management of postoperative pain [6, 7]. As a result, a considerable proportion of patients experience unacceptably high levels of postoperative pain [8–10] with several negative consequences for the patient [11, 12].

National and international surveys have been published analyzing the situation of postoperative pain management [13–18]. It is worth emphasizing that many identified deficits, such as lack of patient information, absence of consensus protocols, incomplete educational programs, or poor adherence to guidelines [14, 18], are more closely related to healthcare organization than to medical issues.

This epidemiological study is a continuation of two previous studies published in 2001 [19] and 2017 [20] that investigated a representative sample of physicians involved in postoperative pain management at hospitals with and without APSs or acute pain management programs (APMPs) across Spain. This survey aims to provide an updated understanding of postoperative pain management and clinical practices in Spain 12 years after the previous survey, evaluating current practice patterns, challenges, and healthcare professionals’ perceptions, and comparing the findings with those reported in the previous survey. Specifically, it focuses on aspects such as organizational structure, patient information, pain assessment, treatment protocols, quality indicators, and training.

## 2. Methods

The Acute Pain Working Group of the Spanish Society of Anesthesiology (SEDAR) designed a questionnaire to determine the current state of postoperative pain management in Spain. In the first phase, four members of the Working Group (GI, SM, JCL, and MPH) conducted a targeted review of current international guidelines and key literature on postoperative pain management. This included the joint guidelines of the American Pain Society, the American Society of Regional Anesthesia and Pain Medicine, and the American Society of Anesthesiologists (APS–ASRA–ASA); the Procedure‐Specific Postoperative Pain Management (PROSPECT) recommendations of the European Society of Regional Anaesthesia and Pain Therapy (ESRA); and the principles of Enhanced Recovery After Surgery (ERAS) programs. Previous national surveys conducted in Spain in 2001 and 2017 were also reviewed to ensure comparability of the results over time. In the second phase, the remaining members of the Working Group (BMS, CD, TF, AMP, NE, and SF) revised the questionnaire until all eleven members agreed on the final version. The final version included 36 items: 24 multiple‐choice questions that had previously been used in 2017 and 12 additional questions that explored the management of postoperative pain in greater depth. Evaluating postoperative pain management across the preoperative, intraoperative, and postoperative phases of pain management, as well as organization and training aspects (Appendix A). Although formal psychometric validation was not performed, the questionnaire was developed based on previously published surveys and current clinical guidelines, supporting its content validity. Furthermore, expert review and the consensus process among all members of the Working Group helped ensure the relevance, clarity, and coverage of the assessed domains.

The Galician Network of Research Ethics Committees approved the study protocol (Registration code: 2023/097, approved on April 14, 2023).

### 2.1. Study Design

We conducted a nationwide, cross‐sectional, observational, multicenter study involving anesthesiologists interested in acute pain management in Spain who worked at public, private, contracted, or state‐funded surgical centers registered in the National Catalog of Hospitals. To comprehensively examine issues surrounding acute pain management in healthcare settings and identify potential areas for further investigation, the research team included both members and nonmembers of the Working Group who were responsible for managing acute pain at their respective hospitals. All participants provided written consent. This manuscript adheres to the applicable CROSS guidelines [21]. No patient‐level clinical data were collected, and all demographic data from participating healthcare professionals were recorded anonymously.

### 2.2. Data Collection and Methods

Data were collected using a structured online questionnaire administered from 9 January to February 26, 2024. The research team designed the questionnaire (Appendix A) based on recommendations contained in guidelines for perioperative pain management [3–5] and piloted and revised the questionnaire while a panel of subject‐matter experts piloted and revised the questionnaire. Thirty‐six items covered the following aspects of pain management among postsurgical patients: (a) structure of pain management and available resources; (b) patient information; (c) assessment and documentation of postoperative pain; (d) treatments administered; (e) indicators and data analysis; (f) training of healthcare personnel; and (g) overall evaluation of pain management. The survey also included demographic questions about hospital affiliation, funding, surgical activity, and geographical location. To be eligible, participants had to practice in one of the specified medical specialties and work at a medical center that performs one or more surgical procedures per month at the time of the survey. There were no restrictions related to the type of surgery or patient characteristics.

### 2.3. Sample Characteristics

A stratified two‐stage balanced sampling strategy was used [22], based on hospital size (< 200 vs. ≥ 200 beds) and funding source (public, private, or mixed/contracted). Thus, in the first stage, responses were grouped according to the hospital where each participant worked. In the second stage, where there were multiple responses from the same hospital with discrepancies that could not be reconciled, the variables were excluded from the analysis.

This approach was selected to ensure representation of the main types of surgical hospitals in Spain and to maintain methodological comparability with the national surveys conducted in 2001 and 2012 [19, 20]. Following this initial selection, participation was voluntary, and responses were obtained through an online survey distributed electronically to healthcare professionals involved in postoperative pain management. All participation in the survey was individual and anonymous.

### 2.4. Survey Administration

The survey was accessible on a website, enabling participants to respond conveniently. Invitations to participate were sent via email, including a link to the survey website with a personal identifier and a disclaimer that the results would be confidential. The questionnaire was distributed among eligible participants who met the inclusion criteria and were available and willing to participate during the study period. Participation was voluntary, and no random selection procedure was applied. Before participating in the survey, a screening question confirming their consent to participate was asked. Approximately 15–20 min were required to complete the questionnaire on the online platform (https://sigmadoscati.tesintegra.net/online.php?pid=SEDAR_2023), and only after all questions were completed online as required could the questionnaire be submitted. Therefore, any questionnaire successfully submitted to the system was considered valid. While the survey was active, the platform identification was stored alongside the survey results and used to prevent duplicate entries. Each respondent could submit a single complete survey.

### 2.5. Study Preparation

Responses were collected on the SEDAR website from 9 January to February 26, 2024. Data were also analyzed according to hospital size (< 200 vs. ≥ 200 beds) and funding source (public, private, or contracted). No specific preparation strategy was implemented before the survey, such as training interviewers or promoting the questionnaire.

### 2.6. Ethical Considerations

Ethical approval for this study (permit number 2023/097) was obtained from the Galician Network of Research Ethics Committees. Confidentiality was ensured by excluding personally identifiable information from the study database and using an internal identification system, together with security tokens, for survey access.

### 2.7. Statistical Analyses

Descriptive statistics were used to summarize the data. The host platform recorded data to calculate the view, participation, or completion rates. Data were stratified according to hospital size (< 200 vs. ≥ 200 beds) and according to whether the hospital had an APS or APMP.

All statistical analyses were performed using the SPSS statistical package (Version 20.0 for Windows).

Continuous variables were summarized using the median and interquartile range (IQR) because several variables had non‐normal distributions. Categorical variables were presented as absolute frequencies, percentages, and 95% confidence intervals (95% CIs). Comparisons between categorical variables were performed using Pearson’s chi‐squared test. Fisher’s exact test was used when the expected frequencies in 2 × 2 contingency tables were less than five. All statistical tests were two‐tailed, and a *p*‐value < 0.05 was considered statistically significant. Missing data were not imputed. Percentages were calculated using the number of available responses for each variable as the denominator. For continuous variables, the mean value of responses from the same institution was calculated. For categorical variables, the modal response was selected as the most representative of the institution.

To prevent duplicate entries, each participant received a unique survey identifier, and the online platform allowed only one submission per respondent. When multiple questionnaires were received from the same institution, responses were aggregated at the hospital level. Continuous variables were summarized using the mean value across respondents from the same institution, whereas categorical variables were consolidated using the modal response. If discrepant responses could not be reconciled, the corresponding variable was excluded from the specific analysis to minimize misclassification bias.

## 3. Results

Approximately 6000 email invitations were sent to SEDAR members (*n* = 4476) and other healthcare professionals (*n* = 1524) (Figure 1). A total of 327 complete questionnaires from 207 centers were returned; multiple responses were received from 76 centers (41 centers provided two responses, and 35 provided three or more responses). Twenty complete questionnaires (17.3%) included paired responses from both private and public settings. After merging these paired responses, a total of 207 unique hospital‐level responses remained.

**FIGURE 1:**
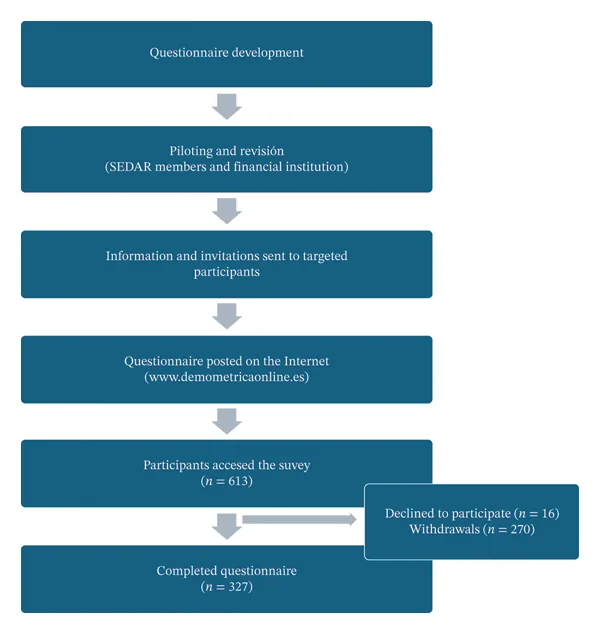
Response distribution and sample characteristics. The 327 participants who completed the questionnaire came from 207 different hospitals. SEDAR: Spanish Society of Anesthesiology, Reanimation, and Pain Therapy.

### 3.1. Respondent Characteristics

Responses were received from 67.9% of the hospitals with ≥ 200 beds (127 of the 187 listed in the national directory of hospitals that perform surgery), while smaller hospitals contributed 23.2% of the responses (80 of the 344 listed in the national directory of hospitals performing surgery). The overall response rate was 38.9%; responses were received from hospitals in all 17 autonomous communities of Spain. Most respondents worked in public hospitals belonging to the National Health System (66.0%); 24.0% worked in private centers, and 10.0% worked in mixed centers. The proportion of hospitals offering university training programs was 83.3%, with 28% providing postgraduate training (at least in medical specialties such as anesthesiology or general and orthopedic surgery). Table 1 shows the surgical complexity category and specialty according to hospital size.

**TABLE 1:** Weekly number of procedures categorized by hospital size, patient hospitalization type, and surgical specialty.

	< 200 beds	≥ 200 beds	Total
	(*n* = 80)	(*n* = 127)	(*n* = 207)
	Median (IQR)	Median (IQR)	Median (IQR)
*Level of complexity and patient hospitalization*			
Hospitalized	68 (15–80)	164 (70–250)	127 (38–200)
Major outpatient	47 (20–64)	123 (40–150)	94 (30–120)
Minor outpatient	40 (17–60)	87 (20–100)	67 (20–97)

*Surgical specialty*			
General	34 (12–45)	39 (20–45)	37 (15–45)
Cardiac	0 (0–0)	4 (0–6)	3 (0–3)
Ear–nose–throat	11 (3–20)	17 (8–20)	14 (5–20)
Gynecologic	18 (3–30)	17 (9–20)	17 (5–24)
Maxillofacial	2 (0–3)	6 (0–10)	5 (0–6)
Neurosurgery	4 (0–4)	6 (0–10)	5 (0–8)
Orthopedic	46 (15–61)	46 (21–60)	46 (19–50)
Plastic	7 (0–7)	10 (0–13)	9 (0–10)
Thoracic	1 (0–1)	4 (0–6)	3 (0–4)
Urologic	22 (4–35)	22 (10–25)	22 (6–20)
Vascular	4 (0–7)	8 (0–15)	6 (0–12)

*Note:* IQR: interquartile range.

### 3.2. Descriptive Results

#### 3.2.1. Pain Management: Structural Organization

Of the 327 physicians who completed the survey, 87.9% worked in the operating room, 24.6% in critical care/emergency departments, 33.8% in pain services, and 21.3% in healthcare management services; many worked in more than one area. Overall, 87.9% of participants were actively involved in postoperative pain management. 70.1% of hospitals with ≥ 200 beds had an institutional APS or APMP, compared with 35.0% of hospitals with < 200 beds (Table 2). Of the large hospitals equipped with APSs, 43.8% provided care for more than 70.0% of surgical patients, while 32.6% provided care for acute pain in nonsurgical patients (Table 3). Full‐time pain physicians were available in 30.3% of hospitals with ≥ 200 beds, and full‐time nurses in 41.6%.

**TABLE 2:** APS, APMP, and human resources availability according to hospital size.

	< 200 beds	≥ 200 beds	Total	*p* value
	(*n* = 28)	(*n* = 89)	(*n* = 117)	
APS or APMP, % (95% CI)	35.0 (25.2–45.8)	70.1 (61.7–77.5)	56.5 (49.7–63.1)	< 0.01
Physicians, full‐time	10.7 (3.1–25.9)	30.3 (21.5–40.4)	25.6 18.4–34.1)	< 0.01
Physicians, part‐time	28.6 (4.5–46.8)	57.3 (46.9–67.4)	50.4 (41.5–59.4)	0.3
Nurses, full‐time	3.6 (0.4–15.5)	41.6 (31.7–51.9)	32.5 (24.5–41.3)	< 0.01
Nurses, part‐time	14.3 (5.0–30.5)	29.2 (20.2–39.2)	25.6 (18.4–34.1)	< 0.01
No specialized staff	57.1 (38.9–74.0)	10.1 (5.1–17.6)	3.4 (1.2–7.9)	0.6

*Note:* APMP: hospital‐wide acute pain management program.

Abbreviations: APS = acute pain service, CI = confidence intervals.

**TABLE 3:** Scope of patient care in hospitals with APS or APMP according to size.

	< 200 beds	≥ 200 beds	Total
	(*n* = 28)	(*n* = 89)	(*n* = 117)
*Percentage of patients with postoperative pain attended, % (95% CI)*			
> 70%	50.0 (32.2–67.8)	43.8 (33.8–54.2)	45.3 (36.5–54.3)
20%–70%	14.3 (5.0–30.5)	31.5 (22.5–41.6)	27.4 (19.9–35.9)
< 20%	14.3 (5.0–30.5)	11.2 (5.9–19.0)	12.0 (7.0–18.8)
DK/NA	21.4	13.5	15.3
Nonsurgical acute pain attended, *n* (%)	28.6 (14.5–46.8)	32.6 (23.5–42.8)	31.6 (23.7–40.4)

*Note:* APMP: hospital‐wide acute pain management program.

Abbreviations: APS = acute pain service, CI = confidence intervals, DK/NA = do not know/no answer.

#### 3.2.2. Postoperative Pain Patient Information

Only 40.2% of hospitals with ≥ 200 beds provided patients with written preoperative information about postoperative pain. This information was usually provided during the pre‐anesthesia evaluation (87.0%). In other cases, this information was provided during the patient’s preoperative consultation with the surgeon (9.0%), on the ward, or in the outpatient surgical area (4.0%).

#### 3.2.3. Postoperative Pain Recording

Over 82.7% of hospitals with 200 or more beds recorded pain assessments, and the percentage was lower in hospitals < 200 beds (71.3%). 81.9% of large hospitals use a specific place for pain assessment in the patient’s chart. In 87.6% of the hospitals where it was documented, it was recorded in medical history alongside vital signs. Additionally, records were computerized in 82.9% of cases (Table 4).

**TABLE 4:** Evaluation and recording of postoperative pain according to hospital size.

	< 200 beds	≥ 200 beds	Total	*p* value
	(*n* = 80)	(*n* = 127)	(*n* = 207)	
	% (95% CI)	% (95% CI)	% (95% CI)	
Postoperative pain assessment is recorded	71.3 (60.7–80.3)	82.7 (75.4–88.5)	78.3 (72.3–83.5)	0.052
The chart contains a place for pain assessment	63.8 (52.9–73.6)	81.9 (74.5–87.8)	74.9 (68.7–80.4)	0.003
Interdepartmental consensus on a pain assessment scale	76.3 (66.1–85.5)	89.0 (82.7–93.5)	84.1 (78.6–88.6)	0.015
Postoperative pain assessment is routine	41.3 (30.9–52.2)	61.4 (52.8–69.5)	53.6 (46.8–60.3)	0.005
Where postoperative pain assessment is recorded	(*n* = 57)	(*n* = 105)	(*n* = 162)	
Patient’s chart, with vital constants	98.2 (92.1–99.8)	87.6 (80.3–92.9)	91.4 (86.3–95.0)	0.022
On a specific data sheet for pain	1.8 (0.2–7.9)	12.4 (7.1–19.7)	8.6 (5.0–13.7)	
Digital recording				
No	26.3 (16.3–38.7)	17.1 (10.9–25.2)	20.4 (14.7–27.1)	0.166
Yes	73.7 (61.3–83.7)	82.9 (74.8–89.1)	79.6 (72.9–85.3)	
Assessment intervals	(*n* = 33)	(*n* = 78)	(*n* = 111)	
Every 4 h	39.4 (24.2–56.4)	12.8 (6.8–21.5)	20.7 (14.0–29.0)	0.015
Every 6 h	12.1 (4.2–26.3)	9.0 (4.1–16.8)	9.9 (5.4–16.5)	
Every 8 h	39.4 (24.2–56.4)	50.0 (39.1–60.9)	46.8 (37.7–56.1)	
Every 12 h	3.0 (0.3–13.3)	1.3 (0.1–5.8)	1.8 (0.4–5.7)	
Every 24 h	6.1 (1.3–18.1)	21.8 (13.8–31.9)	17.1 (11.0–24.9)	
Others	0.0	5.1	3.6 (1.2–8.3)	
Pain management data are analyzed	22.5 (14.4–32.5)	37.9 (29.7–46.6)	31.9 (25.8–38.5)	0.021

*Note:* APMP: hospital‐wide acute pain management program.

Abbreviations: APS = acute pain service, CI = confidence intervals.

#### 3.2.4. Postoperative Pain Assessment

Overall, 89.0% of respondents reported that their hospital used a standardized scale for assessing postoperative pain (76.3% in hospitals with < 200 beds). In hospitals with ≥ 200 beds, the most used tools for postoperative pain assessment were Visual Analog Scales (VAS) (76.4%), Verbal Numerical Scales (62.1%), Faces Pain Rating Scales (35.6%), Verbal Categorical Scales (12.1%), functional activity scoring tools (2.9%), and others (2.3%). 61.4% of hospitals with ≥ 200 beds had designated time intervals for pain assessment (41.3% in small hospitals). The most frequent intervals were every 8 h (50.0%), followed by every 24 h (21.8%), every 4 h (12.8%), and every 6 h (9.0%). Table 4 shows the main results regarding postoperative pain assessment.

#### 3.2.5. Postoperative Pain Treatment Protocol

Table 5 shows that 79.5% of large hospitals had established guidelines for postoperative pain management (65.0% in smaller institutions). The treatment regimen reflected consensus with other hospital departments in 87.1% of cases. Among the hospitals with established protocols for managing postoperative pain, the anesthesiology department was mainly responsible for implementing the treatment protocol (74.3%) and prescribing pain treatment within the hospital (41%). Among the evaluated responses, 74.0% reported that there was no postdischarge pain follow‐up for patients (78.8% in hospitals with < 200 beds).

**TABLE 5:** Postoperative treatment protocol utilization according to hospital size.

	< 200 beds	≥ 200 beds	Total	*p* value
	(*n* = 80)	(*n* = 127)	(*n* = 207)	
	% (95% CI)	% (95% CI)	% (95% CI)	
Postoperative pain protocols in place	65.0 (54.2–74.8)	79.5 (71.9–85.8)	73.9 (67.6–79.5)	0.02
Interdepartmental consensus on protocols	71.2 (57.9–82.1)	87.1 (79.6–92.6)	81.7 (75.0–87.2)	0.015
Protocols target levels of pain intensity	86.5 (75.4–93.8)	89.1 (81.9–94.1)	88.2 (82.4–92.6)	0.64
Protocols established for specific procedures	82.7 (70.8–91.1)	92.1 (85.6–96.2)	88.9 (83.2–93.1)	0.08
Departments applying postoperative pain protocols:				
Anesthesiology	73.1 (60.0–83.7)	74.3 (65.1–82.0)	73.9 (66.5–80.3)	0.197
Surgery	23.1 (13.3–35.8)	15.8 (9.7–23.9)	18.3 (12.8–25.0)	
Postoperative pain service	0.0	6.9 (3.2–13.1)	4.6 (2.1–8.8)	
Other	3.8 (0.8–11.8)	3.0 (0.8–7.7)	3.3 (1.3–7.0)	

*Note:* APMP: hospital‐wide acute pain management program.

Abbreviations: APS = acute pain service, CI = confidence intervals.

#### 3.2.6. Postoperative Pain Indicators and Training

Data from patient records were analyzed to identify opportunities for improving care in 37.9% of hospitals with ≥ 200 beds and 22.5% with < 200 beds. Specific quality indicators for pain management were established in 36.5% of hospitals with ≥ 200 beds and 23.8% with < 200 beds.

Training sessions or meetings were reported in 46.5% of larger hospitals, compared with only 25.7% of smaller hospitals. The training was accredited in 64.0% of hospitals and was mainly provided to the anesthesiology department (75.6%) or APS/APMP staff (41.5%). The sessions mostly centered on nurses (79.0%) and anesthetists (75.3%), while surgeons (55.6%) and specialty trainees (49.4%) were less frequently targeted.

### 3.3. Global Assessment

In large hospitals 55.9% of the participants expressed that the pain was well managed in their hospital, with 49.6% feeling it was managed well and 6.3% feeling it was managed very well, while fewer than 10% of participants rated the pain management in their hospitals as poor (7.2%) or very poor (2.4%).

## 4. Discussion

This survey demonstrates substantial progress in postoperative pain management practices in Spain compared with previous Spanish national surveys conducted in 1997–1998 [19] and 2012 [20] (Figure 2). The response rate was higher in the 2024 survey than in 2012. Among hospitals with ≥ 200 beds. We found an improvement in the availability of APS, full‐time nursing staff, and the engagement of anesthesiologists. The availability of APS increased from 57.7% in 2012 [20] to 70.1%, the proportion of APSs with full‐time nursing staff increased from 25.0% to 41.6%, and the involvement of full‐time anesthesiologists increased from 28.6% to 30.3%.

**FIGURE 2:**
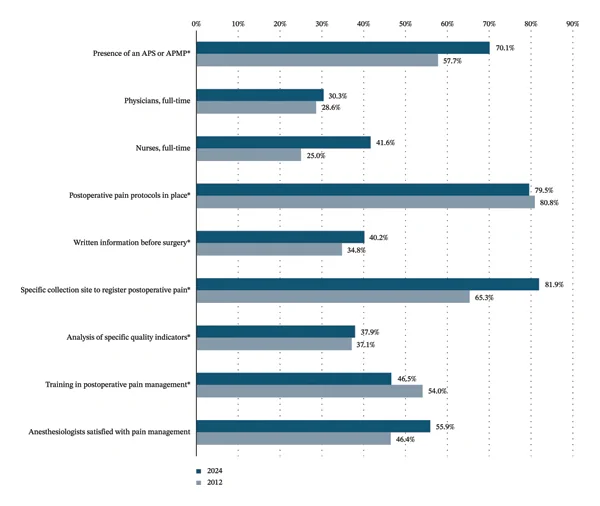
Comparison of responses from the 2024 and 2012^20^ surveys. ∗Hospitals with 200 or more beds. APS: acute pain service; APMP: hospital‐wide acute pain management program.

Smaller hospitals accounted for 23.2% of the responses. This may be explained by the broad distribution of the questionnaire across hospitals of different sizes, including smaller institutions as well as larger referral centers. Their inclusion was intentional, as it allowed the study to capture practices from a wider range of clinical settings and to better reflect real‐world variability. However, the lower proportion of responses from smaller hospitals may also reflect differences in patient volume, available resources, staffing, or engagement with the survey compared with larger centers.

While we identified comparable percentages regarding the existence and implementation of specific postoperative pain protocols to prior surveys (80.8 vs. 79.5%), we noted an enhancement in pain assessment and documentation (65.3% vs. 81.9%), particularly in clinical histories alongside constant vital signs (55.6% vs. 87.6%). In general terms, 12 years earlier, postoperative pain management was deemed inadequate (insufficient, poor, or very poor) for 44.1% of participants [20]. Despite the progress made, we have recognized areas necessitating enhancement in protocol implementation, quality indicators assessment, and training. In contrast to the 2012 survey, we observed an increase in specific qualitative dimensions, including interdepartmental consensus on protocols (74.1% vs. 87.1%) and a reduction in protocols tailored for particular procedures (94% vs. 92.1%), and the implementation of these procedures dependent on expected pain intensity (97.6% vs. 89.1%). Other variables, including the analysis of pain management quality indicators (37.1% vs. 37.9%) and the establishment of meetings or training for postoperative pain management (54.0% vs. 46.5%), were recognized as domains for future enhancement.

Our results align with findings from similar surveys conducted in other countries, although some differences in practice and outcomes are notable. The survey results indicated that 70.1% of large hospitals had an institutional APS or APMP, according to the standards and recommendations for pain services developed in other European countries [23].

These survey results on postoperative acute pain management in Spain reveal critical insights into the current practices, strengths, and areas needing improvement within the Spanish healthcare system, providing a promising outlook for the future. For instance, the increase in the participation of APS/APMP hospitals (67.5% vs. 57.7% in 2012) observed in Spain does follow the trend seen in Germany (81% vs. 36% over the last decade) [16] and the Netherlands (90%) [13]. In contrast to our results, other countries, such as Denmark, found that the number of APS had fallen in 2012 (between 52% and 71%) by 26 compared to previous surveys published in 2006 and 2009. An increase in full‐time staff assignments has also accompanied the rise in the number of APSs and APMPs in Spanish hospitals.

Notably, the proportion of APSs with full‐time nurses increased from 25.0% in 2012 to 41.6% in 2024, while the availability of full‐time staff increased more modestly from 28.6% to 30.3%. A recent audit conducted in Germany showed a consistent improvement in the total staffing levels, with the percentage of physicians and nurses reaching 91% and 25% in 2016, compared to 68% and 8% in earlier surveys, respectively [16]. The effectiveness of a nurse‐based model has already been demonstrated [24, 25], where specialized pain nurses have a key function as mediators and advisors of the nurses in general wards. However, given that APS is viewed as a specialist service offering care to a broader range of patients, the requirement for additional training in pain management and palliative care, as well as at least physician supervision, becomes mandatory [26].

According to our results, in Spain, anesthesiology departments predominantly provided APS (58%), following the trend observed in countries such as the Netherlands (62%) [13]. However, models that included other disciplines or were based on surgery (3.9%) did not have a meaningful impact. Historically, this phenomenon has been attributed to the fact that the responsibilities of an APS were primarily centered around the provision of “catheter service”—the care and supervision of certain invasive procedures [27].

Experts consistently emphasize the importance of explaining to the patient the methods used to manage and evaluate pain [28]. According to our updated results, a higher percentage of hospitals provided written information about postoperative pain (40.2%, up from 34.8% in 2012). These results are consistent with previous surveys conducted in countries such as France [14] and other European countries [18], where 69.3% and 48% of patients either remembered or were systematically provided information about postoperative pain.

However, this survey has some limitations that should be considered. First, our study is designed as a cross‐sectional study, so we cannot draw any clear conclusions about a cause‐effect relationship. Nevertheless, this design offers a suitable structure for a descriptive approach to the concerns discussed in our study. Second, we cannot rule out sampling bias, given limited response rates, particularly for hospitals with < 200 beds (23.2%), even though this rate was higher than the rate of 9.6% 12 years ago [20]. The lower participation rate among hospitals with fewer than 200 beds (23.2%) may reflect the smaller size and lower surgical complexity of these institutions, which frequently lack dedicated APSs or professionals specifically responsible for postoperative pain management. Consequently, the survey topic may have been perceived as less directly relevant in these centers. A similar pattern of lower participation among smaller hospitals was observed in previous national surveys, suggesting a persistent challenge when assessing postoperative pain management in this setting.

Regarding the larger hospitals, although the response rate (67.9%) exceeded the response rate obtained in the 2012 survey (42.4%), it is important to note that our survey targeted responses from all hospitals performing surgery in Spain rather than a sample of them. Another limitation is that the patients were not explicitly interviewed about postoperative pain. Therefore, we are unable to provide evidence that suggests a causal relationship between process indicators and outcomes from the patient’s point of view (reduced pain and satisfaction with treatment). Third, the respondents may not fully represent the target sample. Our study utilized a convenient (nonrandomized) sample of 207 participants that may not be wholly representative of the intended population.

The questionnaire was distributed among eligible participants, and they were available and willing to participate during the study period. Participation was voluntary, and no random selection procedure was applied. Although SEDAR is the scientific society with the largest number of anesthesiologist members, it may not represent all of the country’s anesthesiologists. Therefore, the sample may not be fully representative of the wider target population, which should be considered when interpreting the findings.

A further limitation of this study is the use of a questionnaire that was developed based on previous guidelines and published questionnaires but was not formally validated. Although this approach was intended to ensure that the items were clinically relevant and aligned with existing recommendations, the lack of formal validation may have introduced measurement bias and may limit the reproducibility and comparability of the findings. Future studies should aim to validate the questionnaire through expert review, pilot testing, and psychometric assessment, including reliability, internal consistency, and construct and criterion validity. Whenever possible, questionnaire‐based findings should also be compared with objective measures to further strengthen the validity of the results.

Despite the limitations of our work, this study is the most recent investigation conducted in Spain to assess the current practices, challenges, and perceptions of healthcare professionals in postoperative pain management. Another strength of our survey is that the respondents held positions of authority as heads of APSs or APMPs. Conducting surveys among heads of anesthesiology departments [16] can capture the opinions of physicians with extensive knowledge of all facets of postoperative pain management.

## 5. Conclusion

This survey provides valuable insights into the current state of postoperative acute pain management practices in Spain, highlighting progress and ongoing challenges. These findings show an improvement in comparison to previous work on the following indicators: the digital recording of pain assessments, consensus on scales to use consistently throughout the hospital, and the staff’s satisfaction with their hospital’s pain management. Nevertheless, ongoing efforts are needed to improve professional training, establish standardized treatment protocols, and implement clinical guidelines and specific protocols. These findings may help to understand organizational issues fully and allow them to determine where efforts should be focused to improve current practice, ensuring that all patients receive adequate pain treatment after surgery.

**Nomenclature**
APMPs: Acute pain management programs
APSs: Acute pain services
SEDAR: Spanish Society of Anesthesiology, Reanimation, and Pain Therapy

## Author Contributions

All authors conceived the study and designed the research protocol. Antonio Montes Pérez, Maria A. Perez‐Herrero, and Gustavo Illodo Miramontes interpreted the results. All authors critically reviewed the manuscript.

## Funding

No funding was received for this manuscript. This initiative, which involves research, dissemination, and training projects, has been carried out in collaboration with SEDAR. SEDAR (Acute and Chronic Pain Section) is the main sponsor of the study, with Menarini as a co‐sponsor.

## Disclosure

All authors confirm that they have read the journal’s position on issues involved in ethical publication and affirm that this report is consistent with those guidelines. All authors approved the final version for submission.

## Conflicts of Interest

The authors declare no conflicts of interest.

## Supporting Information

Additional supporting information can be found online in the Supporting Information section.

## Supporting information


**Supporting Information** STROBE checklist.

## Data Availability

The data that support the findings of this study are available from the corresponding author upon reasonable request.

## Appendix A AcuteSpain Questionnaire

*Appendix A1*: Presentation of the AcuteSpain Questionnaire.

The purpose of the AcuteSpain questionnaire is to gather information regarding clinical practice and the care structure of acute pain in anesthesiology departments in Spain, through SEDAR members managing acute pain units. The objective is to further analyze and study this information to better understand the current state of acute pain management in our healthcare environment and to propose future lines of work.

*Appendix A2*: Sponsor of the “AcuteSPain” Study.

The AcuteSPain study is initiated by the Acute Pain Working Group (Acute Pain Working Group) of the SEDAR and is sponsored by the *Spanish Society of Anesthesia, Resuscitation, and Pain* Therapy (SEDAR).

To participate in the AcuteSPain study by completing the AcuteSPain questionnaire, you must first carefully read the attached information and confirm whether you accept the participation conditions.

Informed consent:
  • Yes, read
  • Not read
Do you agree to participate in the AcuteSPain Study (only if the consent has been read):
  • Yes, I agree
  • No, I do not agree

*Appendix A3*: AcuteSPain Questionnaire Questions to Answer.

**Q.1.** In which province is your hospital located? (A list with all Spanish provinces and autonomous cities will be displayed, ordered alphabetically)
**Q.2.** To use other variables included in the National Hospital Catalog, please select the name of your center from the following list (to facilitate the search, the centers are ordered by province).
**Q.3.** Hospital size based on the number of operational inpatient beds:
  1. Less than 200
  2. Between 200 and 600
  3. More than 600
  4. D/K (Don’t know/No answer)
**Q.4.** According to your information: average number of surgical interventions per week:
  1. With hospitalization: number
  2. Major Ambulatory Surgery (MAS): number
  3. Minor Ambulatory Surgery (MAS): number
  4. D/K (for each item)
**Q.5.** According to your information: average number of interventions per week in:
  1. General Surgery
  2. Orthopedic and Trauma Surgery (COT)
  3. Cardiac Surgery
  4. Plastic Surgery
  5. Thoracic Surgery
  6. Vascular Surgery
  7. Gynecological Surgery
  8. Obstetric Surgery (Cesarean section)
  9. Maxillofacial Surgery
  10. Neurosurgery
  11. ENT Surgery (Otorhinolaryngology)
  12. Urological Surgery
  13. D/K (for each item)
**Q.6.** Type of hospital based on functional management:
  1. Public, public management
  2. Public, private management/Privately Contracted
  3. Private, Not Contracted
  4. D/K
**Q.7.** Type of hospital based on training:
  1. University hospital with postgraduate training (at least with MIR in Anesthesiology, General Surgery, COT)
  2. University hospital without postgraduate training
  3. Non‐university hospital
  4. D/K
**Q.8.**What is the department/unit where you currently work? (multiple response)
  1. Clinical practice in the operating room
  2. Clinical practice in critical care units
  3. Clinical practice in pain units
  4. Management
  5. Other (specify)
**Q.9.** Is there an Acute Pain Unit (APU) or a Postoperative Acute Pain Management Program?
  1. Yes
  2. No
  3. D/K (Don’t know/No answer)
**Q.9b.** Does it have administrative recognition?
  1. Yes
  2. No
**Q.9c.** APU’s level of care coverage (multiple response):
  1. Treats all surgical patients
  2. Treats most surgical patients (> 70%)
  3. Treats most surgical patients (50%–70%)
  4. Treats most surgical patients (21%–49%)
  5. Treats most surgical patients (< 20%)
  6. Treats patients with infusion pump/catheter devices
  7. Treats acute pain in nonsurgical patients
  8. D/K (Don’t know/No answer)
**Q.10.** What structure in your hospital addresses the management of acute pain?
  1. Anesthesiology Department
  2. Acute Pain Unit
  3. Chronic Pain Unit
  4. Each professional is responsible for their own patients
  5. Surgical Services
  6. Nursing
**Q.11.** Regarding postoperative pain management, does it cover all operated patients or only some of them?
  1. All
  2. Only certain types of patients
**Q.12.** Regarding postoperative acute pain management, at what point are patients attended to? (multiple response)
  1. During the preoperative period
  2. In the operating room and during transit to the postoperative recovery unit
  3. During their stay in the postoperative recovery unit
  4. During the first 24–48 h of hospital stay, regardless of the location
  5. More than 48 h
  6. Until hospital discharge
**Q.13.** Is there specific personnel for postoperative pain management? (multiple response)
  1. Yes, full‐time physicians
  2. Yes, part‐time physicians
  3. Yes, full‐time nurses
  4. Yes, part‐time nurses
  5. No
  6. Other situations (specify)
  7. D/K (Don’t know/No answer)
**Q.14.** Do you work or participate directly in the management of postoperative pain?
  1. Yes
  2. No

*Appendix A4*: Preoperative Management Questions.

**Q.15.** In your hospital, is the patient provided with any written information about postoperative acute pain before the surgery?
  1. Yes (If yes, proceed to Q.15b and Q.15c)
  2. No
**Q.15b.** If the answer to Q.15 is affirmative, who usually provides this information?
  1. Anesthesiologist
  2. Surgeon
  3. Nurse
  4. Others (specify)
**Q.15c.** If the answer to Q.15 is affirmative, at what point is this written information provided?
  1. Surgical consultation visit (surgery department)
  2. Pre‐anesthetic consultation visit (anesthesiology department)
  3. Hospitalization ward
  4. Others (specify)
**Q.16.** Within the patient’s documentation for the surgical care process, is there a specific section, printed or digital, designated for recording preoperative pain?
  1. Yes
  2. No
**Q.17.** Is there any pain risk assessment scale applied during the preoperative evaluation for the assessment of the risk of persistent postoperative pain?
  1. Yes
  2. No
**Q.17b.** If the answer to Q.17 is affirmative, specify which scale is used:
  1. Categorical Scale (descriptive terms)
  2. Numerical Verbal Scale (NVS)
  3. Visual Analog Scale (VAS)
  4. Faces Scale (indicate for whom: children/elderly)
  5. Functional Activity Scale (FAS)
  6. Others (specify)
**Q.17b.1.** If the answer to Q.17b was “Faces Scale,” what type of patients typically use it?
  1. Children
  2. Elderly
  3. Others (specify)
**Q.18.** Are any strategies implemented during the preoperative period to prevent acute postoperative pain?
  1. Yes (If yes, proceed to Q.18b)
  2. No
**Q.18b.** If the answer to Q.18 is affirmative, select the applicable options (multiple response):
  1. Nonpharmacological treatment (Physical exercise/Psychological support…) (Proceed to Q.18b.1)
  2. Pharmacological treatment (pregabalin/gabapentin/COX inhibitors…) (Proceed to Q.18b.2)
**Q.18b.1.** If the answer to Q.18b included option 1, what type of nonpharmacological therapy is prescribed? (multiple response):
  1. Physical exercise
  2. Physiotherapy
  3. Psychological therapy
  4. Nutritional therapy
  5. Others (specify)
**Q.18b.2.** If the answer to Q.18b included option 2, what type of pharmacological therapy is applied? (multiple response):
  1. Gabapentinoids
  2. COX Inhibitors
  3. Corticosteroids
  4. Paracetamol
  5. Ketamine
  6. Lidocaine
  7. Dexmedetomidine
  8. Magnesium sulfate
  9. Beta‐blockers
  10. Others (specify)

*Appendix A5*: Intraoperative Management Questions.

**Q.19.** Are any strategies implemented during intraoperative management for the prevention/management of postoperative pain?
  1. Yes (If yes, proceed to Q.19b)
  2. No
**Q.19b.** If the answer to Q.19 is affirmative, select the applicable options (multiple response):
  1. Pharmacological therapy (ketamine, lidocaine, magnesium sulfate, dexmedetomidine…) (Proceed to Q.19b.1)
  2. Nonpharmacological therapies (acupuncture, hypnosis, music therapy…) (Proceed to Q.19b.2)
**Q.19b.1.** If the answer to Q.19b included option 1, what type of pharmacological therapy is applied? (multiple response):
  1. Gabapentinoids
  2. COX Inhibitors
  3. Corticosteroids
  4. Paracetamol
  5. Ketamine
  6. Lidocaine
  7. Dexmedetomidine
  8. Magnesium sulfate
  9. Beta‐blockers
  10. Others (specify)
**Q.19b.2.** If the answer to Q.19b included option 2, what type of nonpharmacological therapy is applied? (multiple response):
  1. Hypnosis
  2. Acupuncture
  3. TENS (Transcutaneous Electrical Nerve Stimulation)
  4. Virtual reality glasses
  5. Music therapy
  6. Others (specify)
**Q.20.** Do you have any intraoperative nociception monitoring devices? (multiple response):
  1. Yes (If yes, proceed to Q.20b)
  2. No
**Q.20b.** If the answer to Q.20 is affirmative, what type of intraoperative nociception monitoring devices are available? (multiple response):
  1. ANI (Analgesia Nociception Index)
  2. Cardean
  3. NOL (Nociception Level Index)
  4. qNOX
  5. SPI (Surgical Pleth Index)
  6. Others (specify)
**Q.21.** Are you familiar with the Enhanced Recovery After Surgery (ERAS)/fast track/enhanced recovery protocols?
  1. Yes
  2. No
**Q.22.** Is any ERAS protocol implemented in your center?
  1. Yes (If yes, proceed to Q.22b)
  2. No
**Q.22b.** If the answer to Q.22 is affirmative, in which surgical specialties is it applied? (multiple response):
  1. Vascular
  2. Cardiac
  3. Thoracic
  4. Neurosurgery
  5. Major abdominal surgery
  6. Orthopedic and Trauma Surgery (COT)
  7. Gynecology
  8. Obstetrics (Cesarean section)
  9. Others (specify)

Appendix A6: Postoperative Management Questions.

**Q.23.** In the patient documentation for the surgical care process, is there a specific sectSion designated for recording postoperative pain assessment as a constant (fifth vital sign)?
  1. Yes
  2. No
**Q.24.** Does your hospital use standardized scales for postoperative pain assessment?
  1. Yes (If yes, proceed to Q.24b)
  2. No
**Q.24b.** If the answer to Q.24 is affirmative, select the applicable options:
  1. Categorical Scale (descriptive terms)
  2. Numerical Verbal Scale (NVS)
  3. Visual Analog Scale (VAS)
  4. Faces Scale (children/elderly)
  5. Functional Activity Scale (FAS)
  6. Others (specify)
**Q.24b.1.** If the answer to Q.24b was “Faces Scale,” which type of patients typically use it? (multiple response):
  1. Children
  2. Elderly
  3. Others (specify)
**Q.25.** Are there defined standardized intervals for postoperative pain assessment?
  1. Yes (If yes, proceed to Q.25b)
  2. No
**Q.25b.** If the answer to Q.25 is affirmative, what are these intervals?
  1. 4 h
  2. 6 h
  3. 8 h
  4. 12 h
  5. 24 h
  6. Others (specify)
**Q.26.** Besides being assessed, is postoperative pain evaluation recorded?
  1. Yes (If yes, proceed to Q.26b and Q.26c)
  2. No
**Q.26b.** If the answer to Q.26 is affirmative, where is the postoperative pain assessment recorded? (multiple response):
  1. In the medical record alongside vital signs
  2. In a specific document (Proceed to Q.26b.1)
**Q.26b.1.** If the answer to Q.26b was “in a specific document,” for which types of surgical procedures?
  1. In all surgical procedures, regardless of patient and surgery type
  2. In certain surgical procedures depending on patient and/or surgery type (specify)
**Q.26c.** Is the recording of postoperative pain assessment digitalized?
  1. Yes
  2. No
**Q.27.** Are there protocols for postoperative pain management?
  1. Yes (If yes, proceed to Q.27b through Q.27f)
  2. No
**Q.27b.** If the answer to Q.27 is affirmative, are they agreed upon with other departments?
  1. Yes (Proceed to Q.27b.1)
  2. No
**Q.27b.1.** If the answer to Q.27b is affirmative, is the anesthesiology department responsible for carrying it out?
  1. Yes
  2. No
**Q.27c.** If the answer to Q.27 is affirmative, are these protocols applied based on pain intensity? (e.g., mild, moderate, and severe pain)
  1. Yes
  2. No
**Q.27 d.** If the answer to Q.27 is affirmative, are there specific analgesic protocols for certain surgical procedures?
  1. Yes
  2. No
**Q.27e.** If the answer to Q.27 is affirmative, which department applies the protocol treatment at the end of the surgical intervention?
  1. Anesthesiology
  2. Surgery
  3. Acute Pain Unit
  4. The specific department attending the patient
  5. Others (specify)
**Q.27f.** If the answer to Q.27 is affirmative, which department prescribes pain treatment in the hospitalization ward?
  1. Anesthesiology
  2. Surgery
  3. Acute Pain Unit
  4. The specific department attending the patient
  5. Others (specify)
**Q.28.** How long is the follow‐up of patients with acute postoperative pain conducted?
  1. 24 h
  2. 48 h
  3. 72 h
  4. 72 h
  5. Dependent on patient evolution (specify)
  6. Dependent on type of surgery (specify)
**Q.29.** In your hospital, is any type of pain follow‐up conducted for surgical patients discharged from the hospital?
  1. Yes (If yes, proceed to Q.29b)
  2. No
**Q.29b.** If the answer to Q.29 is affirmative, to which patients is follow‐up provided? (multiple response):
  1. All
  2. Ambulatory Surgery
  3. Selected patients (specify)
**Q.30.** Does the Acute Pain Unit (APU) intervene in analgesic management at hospital discharge?
  1. Yes
  2. No
**Q.31.** Are the data from acute postoperative pain records analyzed to implement improvement actions?
  1. Yes
  2. No
**Q.32.** Are indicators regarding pain management analyzed?
  1. Yes
  2. No
**Q.33.** Are there meetings or courses on postoperative pain management in your hospital?
  1. Yes (If yes, proceed to Q.33b)
  2. No
**Q.33b.** If the answer to Q.33 is affirmative, who are they directed to? (multiple response):
  1. Anesthesiology Department
  2. Surgery Department
  3. Anesthesiology Residents
  4. Residents in general
  5. Nurses
  6. Others (specify)
**Q.33c.** If the answer to Q.33 is affirmative, who conducts them?
  1. Anesthesiology Department
  2. Acute Pain Unit
  3. Chronic Pain Unit
  4. Others (specify)
**Q.33 d.** Are the training activities accredited?
  1. Yes
  2. No
**Q.34.** Do you use social networks for your training?
  1. Yes (If yes, proceed to Q.34b)
  2. No
**Q.34b.** If the answer to Q.34 is affirmative, which ones do you use? (multiple response):
  1. Twitter
  2. Instagram
  3. LinkedIn
  4. Others (specify)
**Q.35.** Do you consider that your clinical practice is supported by scientific evidence?
  1. Yes, completely
  2. Yes, mostly
  3. Yes, only partially
  4. I don’t know
**Q.36.** Lastly, we request a global assessment: How do you perceive the management of postoperative pain in your hospital?
  1. Very well
  2. Well
  3. Fair
  4. Poor
  5. Very poor
