# Supplementary material for: Evolution of Management of Postoperative Pain in Spain: A Nationwide Survey of Organizational Structure and Clinical Practice
Source: Pain Res Manag. 2026 Nov 2;2026:5391303. doi: 10.1155/prm/5391303 (PMC13728333; doi:10.1155/prm/5391303)
Supplement: Supplementary file 1 [file PRM-2026-5391303-s001.docx]

STROBE Statement—checklist of items that should be included in reports of observational studies

|  | Item No. | Recommendation | Page  No. | Relevant text from manuscript |
| --- | --- | --- | --- | --- |
| **Title and abstract** | 1 | (*a*) Indicate the study’s design with a commonly used term in the title or the abstract | 4 | A cross-sectional study |
|  |  | (*b*) Provide in the abstract an informative and balanced summary of what was done and what was found | 4 – 5 | Twelve years later, our study shows, there has been an increase in the percentage of centers with APS or APMP, the proportion of nurses with full-time dedication, and the assessment and documentation of pain. However, the implementation of protocols, pain management training, and the analysis of pain-related indicators have worsened. |
| Introduction | | | |  |
| Background/rationale | 2 | Explain the scientific background and rationale for the investigation being reported | 7 | The present study builds on two earlier investigations conducted in 2001 (Puig et al. 2001) and 2017 (Montes et al. 2017), which surveyed representative samples of physicians working in Spanish hospitals with and without acute pain services (APSs) or acute pain management programs (APMPs). |
| Objectives | 3 | State specific objectives, including any prespecified hypotheses | 7 | The objective of this survey is to offer an updated overview of postoperative pain management practices in Spain more than a decade later, by assessing current clinical approaches, perceived challenges, and professional perspectives. |
| Methods | | | |  |
| Study design | 4 | Present key elements of study design early in the paper | 7 | We conducted a nationwide, cross-sectional, observational, multicenter study. |
| Setting | 5 | Describe the setting, locations, and relevant dates, including periods of recruitment, exposure, follow-up, and data collection | 7 – 8 | The study involving anesthesiologists interested in acute pain management in Spain, whether they are public, private, or contracted or state-funded surgical entities registered in the National Catalog of Hospitals. |
| Participants | 6 | (*a*) *Cohort study*—Give the eligibility criteria, and the sources and methods of selection of participants. Describe methods of follow-up  *Case-control study*—Give the eligibility criteria, and the sources and methods of case ascertainment and control selection. Give the rationale for the choice of cases and controls  *Cross-sectional study*—Give the eligibility criteria, and the sources and methods of selection of participants | 8 | The research team included both members and non-members responsible for managing the acute pain at the hospital where they assist. |
|  |  | (*b*) *Cohort study*—For matched studies, give matching criteria and number of exposed and unexposed  *Case-control study*—For matched studies, give matching criteria and the number of controls per case |  |  |
| Variables | 7 | Clearly define all outcomes, exposures, predictors, potential confounders, and effect modifiers. Give diagnostic criteria, if applicable | 8 | The information was collected through a structured questionnaire with an online survey conducted from 9th January to 26th February 2024. |
| Data sources/ measurement | 8* | For each variable of interest, give sources of data and details of methods of assessment (measurement). Describe comparability of assessment methods if there is more than one group | 8 | Thirty-one items covered the following aspects of pain management among postsurgical patients: a) Structure of pain management and available resources, b) Patient information, c) Assessment and documentation of postoperative pain, d) Treatments administered, e) Indicators and data analysis, f) Training of healthcare personnel, and g) Overall evaluation of pain management. |
| Bias | 9 | Describe any efforts to address potential sources of bias | 11 | When we received multiple responses for continuous variables from a single institution, we conducted an analysis of the mean scores. In the case of multiple responses for categorical variables, where it was not feasible to determine the correct answer, we excluded the response. |
| Study size | 10 | Explain how the study size was arrived at | 7-8 | We conducted a nationwide, cross-sectional, observational, multicenter study involving anesthesiologists interested in acute pain management in Spain related to the study's objectives, whether they are public, private, or contracted or state-funded surgical entities registered in the National Catalog of Hospitals. |

Continued on next page

| Quantitative variables | 11 | Explain how quantitative variables were handled in the analyses. If applicable, describe which groupings were chosen and why | 8 | Thirty-one items covered the following aspects of pain management among postsurgical patients: a) Structure of pain management and available resources, b) Patient information, c) Assessment and documentation of postoperative pain, d) Treatments administered, e) Indicators and data analysis, f) Training of healthcare personnel, and g) Overall evaluation of pain management. The survey also included demographic questions about hospital affiliation, funding, surgical activity, and geographic locations. |
| --- | --- | --- | --- | --- |
| Statistical methods | 12 | (*a*) Describe all statistical methods, including those used to control for confounding | 11 | Continuous variables were described by the mean and standard deviation. Where appropriate, categorical variables were presented as percentages with a 95% confidence interval (95% CI). To compare categorical variables, we employed the chi-square test. However, if we anticipated frequencies below 5 for variables in 2x2 contingency tables, we opted for the Fisher exact test. Results with values of p< 0.05 were considered statistically significant. |
|  |  | (*b*) Describe any methods used to examine subgroups and interactions | 11 | When we received multiple responses for continuous variables from a single institution, we conducted an analysis of the mean scores. In the case of multiple responses for categorical variables, where it was not feasible to determine the correct answer, we excluded the response. |
|  |  | (*c*) Explain how missing data were addressed | 11 | Missing data was not imputed. |
|  |  | (*d*) *Cohort study*—If applicable, explain how loss to follow-up was addressed  *Case-control study*—If applicable, explain how matching of cases and controls was addressed  *Cross-sectional study*—If applicable, describe analytical methods taking account of sampling strategy |  |  |
|  |  | (*e*) Describe any sensitivity analyses |  |  |
| Results | | | | |
| Participants | 13* | (a) Report numbers of individuals at each stage of study—eg numbers potentially eligible, examined for eligibility, confirmed eligible, included in the study, completing follow-up, and analysed | 11 | Approximately 6000 email invitations were distributed among SEDAR members (n=4476) and health professionals (n=1524) (Figure 1). Three hundred twenty-seven complete questionnaires from 207 centers were returned; we received multiple responses from 76 centers. Twenty complete questionnaires (17.3%) included double responses from both private and public settings. After merging all the double responses into single responses, the total number of single responses was 207. |
|  |  | (b) Give reasons for non-participation at each stage |  |  |
|  |  | (c) Consider use of a flow diagram | 11 | Figure 1 |
| Descriptive data | 14* | (a) Give characteristics of study participants (eg demographic, clinical, social) and information on exposures and potential confounders |  |  |
|  |  | (b) Indicate number of participants with missing data for each variable of interest |  |  |
|  |  | (c) *Cohort study*—Summarise follow-up time (eg, average and total amount) | 11 | Three hundred twenty-seven complete questionnaires from 207 centers were returned; we received multiple responses from 76 centers. Twenty complete questionnaires (17.3%) included double responses from both private and public settings. |
| Outcome data | 15* | *Cohort study*—Report numbers of outcome events or summary measures over time |  |  |
|  |  | *Case-control study—*Report numbers in each exposure category, or summary measures of exposure |  |  |
|  |  | *Cross-sectional study—*Report numbers of outcome events or summary measures | 12 | Responses were obtained from hospitals with ≥200 beds at a rate of 67.9% (127/187), while smaller hospitals contributed 23.2% of the replies (80/344). The overall response rate was 38.9%, responses were received from hospitals in the 17 autonomous communities that make up the Spanish state. |
| Main results | 16 | (*a*) Give unadjusted estimates and, if applicable, confounder-adjusted estimates and their precision (eg, 95% confidence interval). Make clear which confounders were adjusted for and why they were included | Tables 2, 3, 4, 5 |  |
|  |  | (*b*) Report category boundaries when continuous variables were categorized |  |  |
|  |  | (*c*) If relevant, consider translating estimates of relative risk into absolute risk for a meaningful time period |  |  |

Continued on next page

| Other analyses | 17 | Report other analyses done—eg analyses of subgroups and interactions, and sensitivity analyses |  |  |
| --- | --- | --- | --- | --- |
| Discussion | | | | |
| Key results | 18 | Summarise key results with reference to study objectives |  |  |
| Limitations | 19 | Discuss limitations of the study, taking into account sources of potential bias or imprecision. Discuss both direction and magnitude of any potential bias |  |  |
| Interpretation | 20 | Give a cautious overall interpretation of results considering objectives, limitations, multiplicity of analyses, results from similar studies, and other relevant evidence |  |  |
| Generalisability | 21 | Discuss the generalisability (external validity) of the study results |  |  |
| Other information | |  | | |
| Funding | 22 | Give the source of funding and the role of the funders for the present study and, if applicable, for the original study on which the present article is based |  |  |

*Give information separately for cases and controls in case-control studies and, if applicable, for exposed and unexposed groups in cohort and cross-sectional studies.

**Note:** An Explanation and Elaboration article discusses each checklist item and gives methodological background and published examples of transparent reporting. The STROBE checklist is best used in conjunction with this article (freely available on the Web sites of PLoS Medicine at http://www.plosmedicine.org/, Annals of Internal Medicine at http://www.annals.org/, and Epidemiology at http://www.epidem.com/). Information on the STROBE Initiative is available at www.strobe-statement.org.
